# Influence of ns-Laser Cleaning Parameters on the Removal of the Painted Layer and Selected Properties of the Base Metal

**DOI:** 10.3390/ma13235363

**Published:** 2020-11-26

**Authors:** Xinyan Li, Dan Wang, Juming Gao, Weiwei Zhang, Canyang Li, Nianzheng Wang, Yucheng Lei

**Affiliations:** 1 School of Materials Science and Engineering, Jiangsu University, Zhenjiang 212013, China; 2221805084@stmail.ujs.edu.cn (X.L.); 2221905013@stmail.ujs.edu.cn (J.G.); 2211805032@stmail.ujs.edu.cn (W.Z.); 3170704099@stmail.ujs.edu.cn (C.L.); yclei@ujs.edu.cn (Y.L.); 2 Key Laboratory of High-end Structural Materials of Jiangsu Province, Jiangsu University, Zhenjiang 212013, China; 3 Key Laboratory of Agricultural Machinery Equipment Remanufacturing Technology of Jiangsu Province, Jiangsu University, Zhenjiang 212013, China; 4 Suzhou Dvellk Photo-Electricity Technology Co., Ltd., Suzhou 215000, China; nz.wang@dvellk.com

**Keywords:** ns-laser cleaning, paint layer, roughness, element distribution, fine grain layer

## Abstract

The removal of the surface paint of Q345 (Gr·B) steel, as well as microstructure and hardness of the cleaned surface were investigated. The laser source used in this study is a nanosecond pulsed Gaussian light source. The surface morphology and microstructure were characterized by a scanning electron microscope and electron back-scattered diffraction. A hardness test was used for capturing variations of the parameter of the cleaned region in comparison to the base metal. The results show that when the X-scanning speed was 1500 mm/s and Y-moving speeds was 7 mm/s during ns-laser cleaning, respectively, the cleaned surface was relatively flat and there was only a few small residual paint. In addition, the contents of Fe and C elements on the cleaned surface reached to 89% and 9%, respectively. Moreover, the roughness was the lowest of 0.5 μm through the observation of the three-dimensional topography. In addition, a fine grain layer appeared on the cleaned surface after laser cleaning at the X-scanning speeds of 500 mm/s and 1000 mm/s. The maximum hardness of the fine grain layer was more than 400 HV, higher than the base metal.

## 1. Introduction

Structural steels, having a high fracture toughness, beneficial fundamental mechanical properties, and good weldability, are widely used in manufacturing ships, airplanes, automobiles, bridges, railways, and oil and gas pipeline industries [1,2,3]. For the purpose of preventing materials from rusting and improving the service life of structural parts, the surface of components is always covered in paint layer. Even so, some surfaces still need to be overhauled over a period of time, since part of the paint layers could fall off. Thus, it is necessary to remove the paint layer in order to facilitate the inspection and reinstall the paint layer [4].

At present, the commonly used methods to clean the surface paint layer include mechanical process [5], chemical stages [6], and ultrasonic methods [7] etc. Shot blasting is a common type of operation in mechanical equipment, but if the flow of impacted abrasive particles is not controlled, it will cause certain damage to the surface [5,8,9]. Pickling is one of the main methods in chemical cleaning. Its disadvantage is that the substrate is easily corroded and has certain pollution to the environment [6]. During the pickling process, the volatilization of hydrochloric acid will cause physical damage to the operator, and improper disposal of waste liquid could cause environmental pollution. In a case of ultrasonic technique, due to the limitation of the size and shape of the workplace, it is difficult to be applied it for a wide range of structures.

In comparison with typical cleaning methods, laser manner, at the following advantages such as indirect contact, environmental protection, and pollution-free, can be applied in automatic process, giving the high quality of surface [10,11,12,13]. Laser cleaning with great flexibility and precision a wide range of materials. The ultrashort laser source could offer high laser intensity and provide high precision of the microdimple geometries. A heat-affected volume would occur when the materials were ablated by the ultrashort pules. Ultrashort-laser are usually used for precision micromachining, such as vision-correction surgery. Due to the dominant multiphoton absorption and ionisation processes, the ultrashort-laser cleaning efficiency is less sensitive to the chemical composition of the contaminants, when compared with ns-lasers. In addition, ns-laser could remove large chunks of material through shock-wave destruction and damage the surrounding material [14,15,16]. Kumar et al. [17] estimated the threshold flux of laser peeling epoxy coating basing on the material properties. Moreover, an appropriate scanning speed process window was established to remove the paint layer. Zhao et al. [18] studied the effect of laser scanning speed, line spacing, laser power, and pulse frequency on the cleaning quality of the 50 μm thick polyacrylic resin primer layer on LY12 aluminum alloy plate. The results have shown that the appropriate scanning speed and pulse frequency play an important role in improving the cleaning quality. The surface paint of aluminum-based materials was removed through changing laser power density, focus length, interaction time, absorption coefficient and other process variables [19]. In addition, a simple regression linear model was established during paint stripping. Watkins et al. [20] systematically explained the cleaning mechanism and summarized six types: ablation, vibration, vaporization, light pressure, vaporization pressure, and plasma burst. Moreover, some researches also have shown that the mechanical properties of the surface of the materials could enhance after laser cleaning. Shamsujjoha et al. [21] found that the metal matrix melted and re-solidified during the process of laser removing paint, and further the surface hardness of materials increased. Shi et al. [22] pointed out that laser cleaning could effectively remove the oxide film on the aluminum alloy surface and also easily formed a hardened layer on the surface of the Al matrix. However, there is still scant research on the quantitative analysis of the removed paint surface by changing both laser Y-moving speed and X-scanning speed during laser cleaning.

In this paper, the paint layer on the Q345 steel is employed for the laser cleaning technology. The effect of laser X-scanning speed and Y-moving speed on the surface morphology, element distribution, and roughness is investigated. Furthermore, the hardness profile is studied.

## 2. Experimental Procedure

### 2.1. Materials

**Figure 1:**
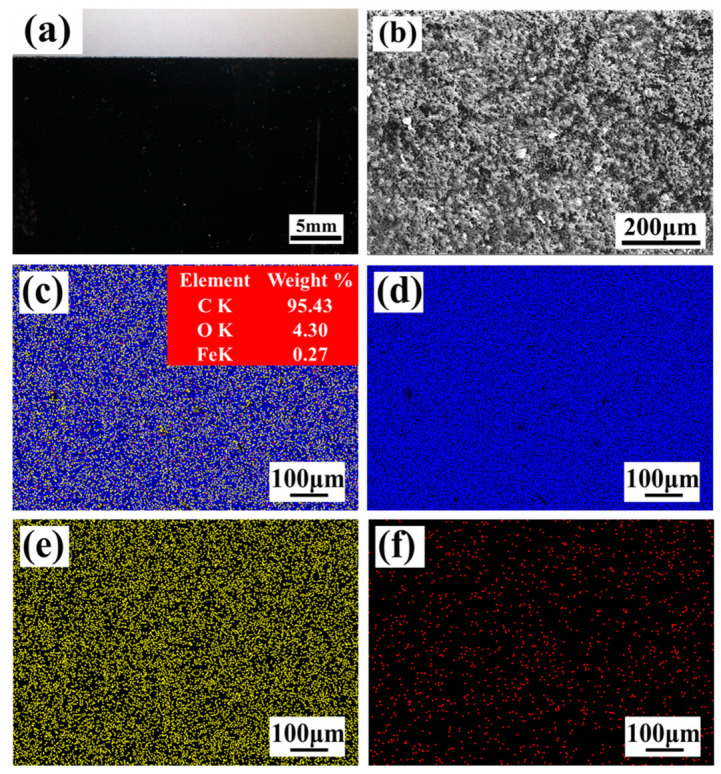
Morphology and EDS mapping of the black paint layer on the surface of Q345 steel. (**a**) OM; (**b**) SEM. (**c**) Overlay elements and percentage content; (**d**) C element; (**e**) O element; (**f**) Fe element.

The material used was Q345 (Chinese standard), same as Gr. B (Europe standard). The chemical composition is shown in Table 1. The surface of Q345 steel in a form of sheet is shown in Figure 1. The optical microscope (OM) image of the surface covered by the black paint layer is presented in Figure 1a, and the paint layer was distributed uniformly and densely as it was investigated by the scanning electron microscope (SEM, FEI, Hillsboro, OR, USA), Figure 1b. Figure 1 illustrates the result of energy disperse spectroscopy (EDS, FEI, Hillsboro, OR, USA) on the black paint layer. The content and distribution of elements are visible in Figure 1c. The main elements of the black paint layer were C, as Figure 1c,d indicate. Moreover, a small amount of O was distributed on the paint layer and the content of Fe was negligible because of the appearance of the covered black paint layer, as of Figure 1e,f.

### 2.2. Experiment

**Figure 2:**
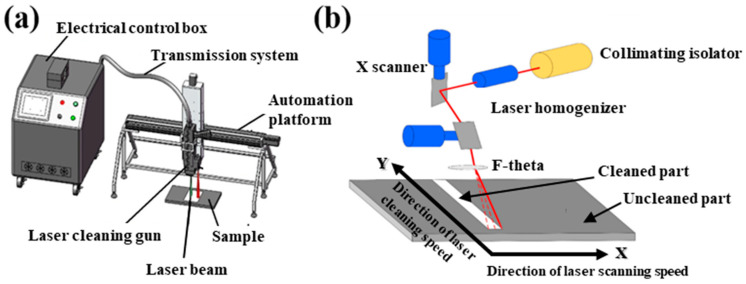
Laser cleaning platform system. (**a**) automatic laser cleaning equipment; (**b**) schematic diagram of cleaning operation [22].

Figure 2 shows the ns-laser cleaning experimental system. The device is mainly composed of a pulsed fiber laser (Oling 200 W MOPA, Orion Laser Technology, Shenzhen, China), a hand-held laser cleaning gun, an automatic bench, an electrical control box, and a transmission system for realizing automatic and quantitative cleaning, Figure 2a. Figure 2b focuses the schematic diagram of laser cleaning operation. The materials surface contaminates, like residual paint layer, are melted and steamed by the radiation of a high frequency pulsed fiber. X-direction represents the direction of laser X-scanning speed to achieve one-dimensional linear motion, and Y-direction is defined as the direction of laser Y-moving speed to achieve two-dimensional surface cleaning by combining the movement of laser scanning.

The main process parameters of laser cleaning are reported in Table 2. The pulsed fiber laser with a wavelength of 1064 nm was employed, and full rated power of 100 W was used. During cleaning, the spot diameter condensed to the material surface through the field lens is about 0.04 mm. The laser X-scanning speed and Y-moving speed were changed from 500 mm/s to 2000 mm/s, and from 5 mm/s to 11 mm/s, respectively. After cleaning, a stereo-microscope, metallographic microscope, SEM, EDS, and electron back-scattered diffraction (EBSD) were used to observe the morphology, distribution of elements and microstructure of the surface and cross-section of the cleaned material. A laser scanning confocal microscope (OLS4100, Olympus, Tokyo, Japan) was used to perform the characterization of three-dimensional morphology and measure surface roughness. A FM-ARS900 automatic micro hardness tester (FUTURE-TECHCORP, Tokyo, Japan) was used for determining the hardness on the cross-section of the sample.

## 3. Results and Discussion

### 3.1. Surface Morphology of the Cleaned Material

In order to investigate the effect of speed parameter on Q345 surface paint laser removal, the surface morphology of the cleaned material should be observed by means of OM and SEM.

#### 3.1.1. The Effect of Laser X-Scanning Speed

**Figure 3:**
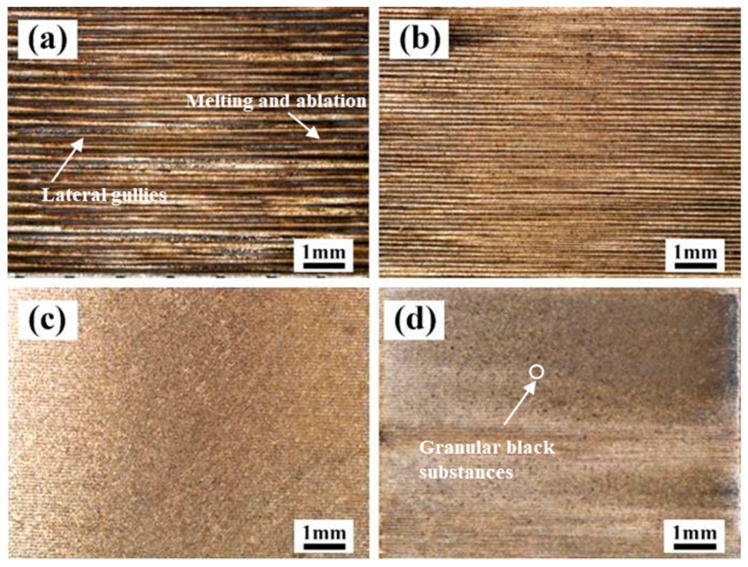
The effect of laser X-scanning speed on the macro morphology of the cleaned surface of Q345 steel at a laser Y-moving speed of 7 mm/s. (**a**) 500 mm/s; (**b**) 1000 mm/s; (**c**) 1500 mm/s; (**d**) 2000 mm/s.

The effect of the laser X-scanning speed on the macro morphology of the cleaned surface of the Q345 at a laser Y-moving speed of 7 mm/s is presented in Figure 3. When the X-scanning speed was of 500 mm/s, plenty of the lateral gullies appeared on the surface and the black between the gullies seemed to be the residual paint layer. Because of the Gaussian distribution of this laser intensity, the intensity of the middle of laser spot is higher than that of the sides. In addition to the relatively high heat input, the difference of the intensity between the middle and the sides of the laser spot could be enlarged, leading to the formation of a deep trace when a laser scanned the surface of the Q345 steel. It can also be found that the appearances of the melting and ablation were serious in selected areas, resulting in a great damage to the material, as shown in Figure 3a. When the X-scanning speed reached 1000 mm/s, the traces of the lateral gullies was reduced. It is worth nothing that the widths of the gullies were also minimized and the distribution became relatively denser. When the X-scanning speed increased up to 1500 mm/s, the surface of the Q345 steel was completely exposed and relatively smooth. Moreover, the traces of lateral ravines were almost invisible, and also the black residue was less. Further increasing the speed up to 2000 mm/s, there were no traces of lateral ravines, but the granular black substances were observed on the surface.

**Figure 4:**
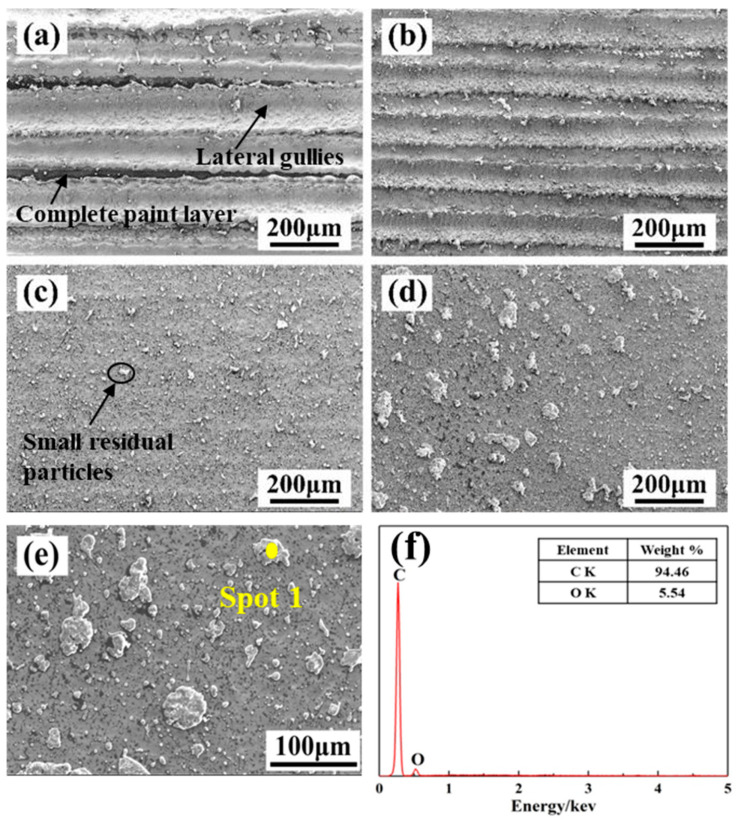
The effect of laser X-scanning speed on the micro-morphology (SEM and EDS) of the paint layer on the surface of Q345 steel when the laser Y-moving speed is 7 mm/s. (**a**) 500 mm/s; (**b**) 1000 mm/s; (**c**) 1500 mm/s; (**d**) 2000 mm/s; (**e**) enlarged SEM image; (**f**) the result of the point scanning of the residual particles.

Figure 4 shows the effect of the laser X-scanning speed on the SEM morphology of the cleaned surface, and the SEM images in Figure 4 are corresponding to those in Figure 3. When the laser X-scanning speed was equal to 500 mm/s, the depth and width of the trace of the lateral gullies were relatively deeper and wider, about 190 μm wide. In addition, it can be found that a complete paint layer still remained in the edge area of the scan, as shown in Figure 3a and Figure 4a. At the low X-scanning speed, the radiation per unit area is more on the surface of the material, leading to a rise in the temperature. Once the temperature is much higher than the melting points of the paint layer and the base material, it will cause the melting and vaporization of the substrate, causing surface damage [23,24,25]. When the X-scanning speed of the material was 1000 mm/s, the gullies became shallower and narrower, just 90 μm wide, as shown in Figure 4b. As the X-scanning speed increased further, the traces of the gully disappeared gradually, and a relatively flat and smooth metal substrate was exposed as a whole. In addition, most of the paint on the surface of the material was removed, and there were only a few small residual particles, as shown in Figure 4c. With the X-scanning speed of 2000 mm/s, the size of the residual particles on the surface increased.

In order to confirm the specific composition of the particle, EDS was performed. Figure 4e shows the results of the EDS of the residual particles on the cleaned surface of the Q345 steel at a Y-moving speed of 7 mm/s and a X-scanning speed of 2000 mm/s. Figure 4e indicates an enlarged SEM image of the residual particles in Figure 4d. It can be seen that the residual particles were uniformly distributed on the surface of the material. The main elements of the residual paint layer are C and O that are the same as that of the paint layer, as shown in Figure 4f. Thus, it can be understood that if there are plenty of large residual paint distributing on the cleaned surface, the cleaning effect is relatively poor. In contrast, the dispersed distribution of small residual paint or negligible residual paint occurred as a result of a relatively better cleaning effect. It is evident that at a low X-scanning speed, the paint on the surface of the material could be completely peeled off, or maybe the laser can damage the material. In addition, the paint could not be removed well at a high X-scanning speed. Appropriate X-scanning speed (1500 mm/s) has a significant impact on the realization of laser cleaning of the surface paint layer of the material.

#### 3.1.2. The Effect of Laser Y-Moving Speed

**Figure 5:**
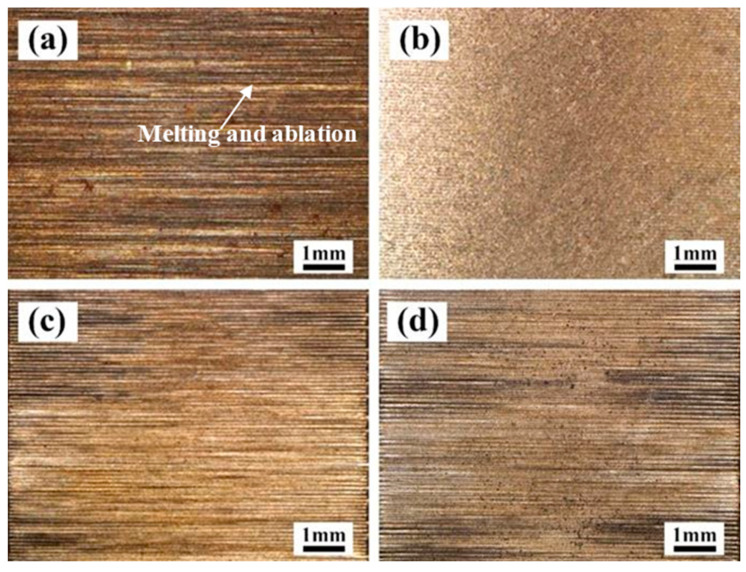
The effect of laser Y-moving speed on the macro morphology of the cleaned surface. (**a**) 5 mm/s; (**b**) 7 mm/s; (**c**) 9 mm/s; (**d**) 11 mm/s at X-scanning speed of 1500 mm/s.

The effect of laser Y-moving speed on the macro morphology of the cleaned Q345 steel surface at a laser X-scanning speed of 1500 mm/s are presented in Figure 5. When the Y-moving speed was of 5 mm/s, it could be found that some areas of the material were melted and ablated seriously, and the ablated dark brown appeared on the surface, Figure 5a. When the Y-moving speed reached 7 mm/s, the paint was removed well and the surface was relatively flat in, as shown in Figure 5b. If, the Y-moving speed reached 9 mm/s and 11 mm/s, some residual paints were observed between the gullies of the surface, as shown in Figure 5c,d.

**Figure 6:**
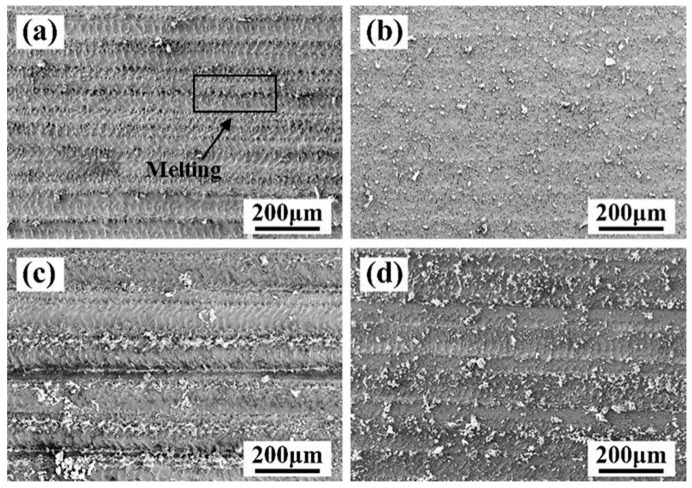
The effect of laser Y-moving speed on the micro-morphology of the cleaned surface. (**a**) 5 mm/s; (**b**) 7 mm/s; (**c**) 9 mm/s; (**d**) 11 mm/s at laser X-scanning speed of 1500 mm/s.

The effect of the laser Y-moving speed on the SEM morphology of the cleaned surface, and the SEM images (Figure 6) are corresponding to those in Figure 5. When the Y-moving speed has taken 5 mm/s, the material was melted seriously and the trace of the lateral gullies was observed, as shown in Figure 6a. As the Y-moving speed increased up to 7 mm/s, the surface was relatively flat and there were only a few small residual particles, as shown in Figure 6b. When the Y-moving speed was more than 9 mm/s, the action time of the laser radiation on the surface became short, leading to the appearance of the more remained paint layer, as shown in Figure 6c,d.

### 3.2. Distribution of Element on the Surface of the Cleaned Material

In order to further confirm the cleaning effect, it is also necessary to characterize the element analysis, such as the contents of Fe (main element in base metal) and C (main element in paint), on the cleaned surface.

#### 3.2.1. The Effect of Laser X-Scanning Speed

**Figure 7:**
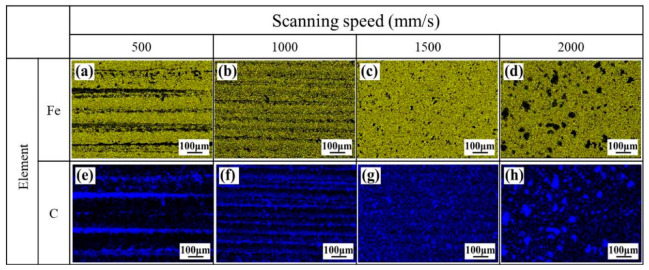
The effect of laser X-scanning speed on the distribution of the elements of the cleaned surface. (**a**) Fe, at 500 mm/s; (**b**) Fe, at 1000 mm/s; (**c**) Fe, at 1500 mm/s; (**d**) Fe, at 2000 mm/s; (**e**) C, at 500 mm/s; (**f**) C, at 500 mm/s; (**g**) C, at 500 mm/s; (**h**) C, at 500 mm/s.

**Figure 8:**
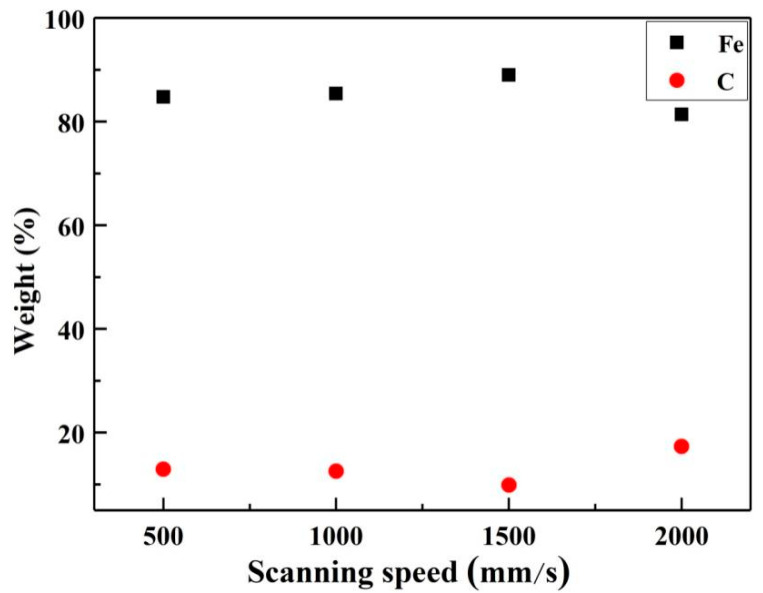
The effect of laser X-scanning speed on the contents of the elements of the cleaned surface.

Figure 7 reports the results of the map scanning of the EDS of the cleaned surface at different X-scanning speeds when the Y-moving speed is 7 mm/s. By comparison of the distribution of Fe element, at the relatively low X-scanning speed, like 500 mm/s and 1000 mm/s, lots of band-shaped traces with light yellow could be observed, as shown in Figure 7a,b. With increasing the X-scanning speed further, the band-shaped traces disappeared and light-yellow points distributed on the surface, as shown in Figure 7c,d. In case of the distribution of C element, some band-shaped traces with deep blue distributed loosely on the surface at the X-scanning speeds of 500 mm/s and 1000 mm/s, as shown in Figure 7e,f. However, the deep blue appeared in terms of point distributions, and the points were relatively small at a X-scanning speed of 1500 mm/s, comparing with that of 2000 mm/s, as shown in Figure 7g,h. In addition, when the X-scanning speed increased from 500 mm/s up to 1500 mm/s, the Fe content increased from 84% to the peak value of about 89%; the C content decreased from 13% to the lowest value of about 9%, as can be seen in Figure 8.

When the X-scanning speed was relatively small, the scanning path with the band-shaped distribution could be observed easily. Most of the paints were removed, however, some remained in the edge area, leading to the occurrence of low content of Fe and high content of C. Meanwhile, the increased X-scanning speed can reduce the time of radiation per unit area. Thus, the scanning path disappeared and Fe elements distributed uniformly on the cleaned surface. The X-scanning speed further increased and the radiation per unit time was insufficient, leading to the appearance of large residual paint.

#### 3.2.2. The Effect of Laser Y-Moving Speed

**Figure 9:**
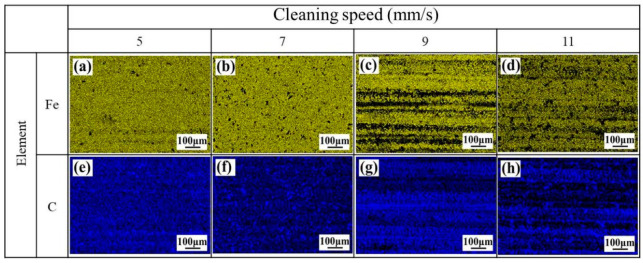
The effect of laser Y-moving speed on the distribution of the elements in the cleaned surface during laser cleaning. (**a**) Fe, at 5 mm/s; (**b**) Fe, at 7 mm/s; (**c**) Fe, at 9 mm/s; (**d**) Fe, at 11 mm/s; (**e**) C, at 5 mm/s; (**f**) C, at 7 mm/s; (**g**) C, at 9 mm/s; (**h**) C, at 11 mm/s.

**Figure 10:**
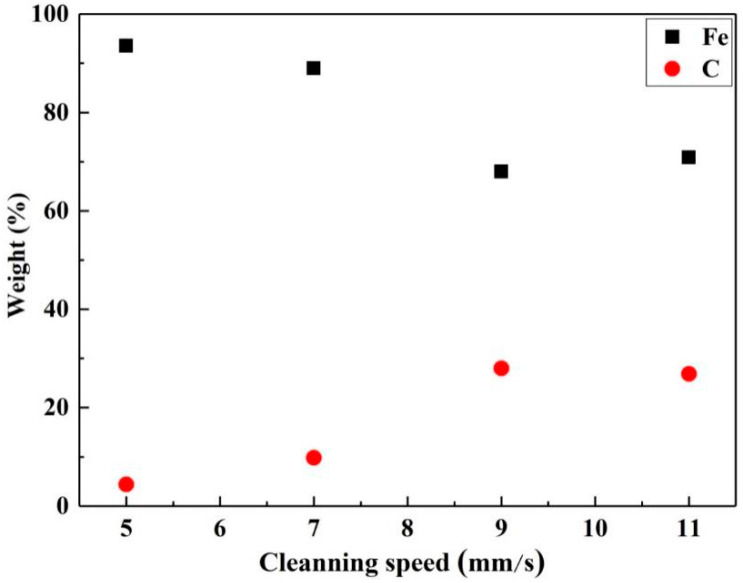
The effect of laser Y-moving speed on the element content in the cleaned surface.

Figure 9 presents the results of the map scanning of the EDS of the cleaned surface at different Y-moving speeds when the X-scanning speed is 1500 mm/s. By comparison of the distribution of Fe element, at relatively low Y-moving speed, like 5 mm/s and 7 mm/s, light yellow appeared in terms of point distributions, as shown in Figure 9a,b. With the Y-moving speed increased, yellow band-shaped traces appeared, as shown in Figure 9c,d. In case of the distribution of C element, the deep blue appeared in terms of point distributions, and the points were relatively sparse at a X-scanning speed of 7 mm/s, compared with that of 5mm/s, could be observed in Figure 9e,f. However, some band-shaped traces with deep blue distributed loosely on the surface at the Y-moving speeds of 9 mm/s and 11 mm/s, (Figure 9g,h). In addition, when the Y-moving speed increased from 5 mm/s up to 11 mm/s, the Fe content decreased from 93% to the valley value of about 68% and kept stable, while the C content increased to from 6% to 27% and remained unchanged, as can be seen in Figure 10. The increased Y-moving speed can make the time of laser action on the material surface short, thus the residual paint might be melted but not be vaporized, the cleaning path started to appear, and the band-shaped traces of C and Fe elements distributed on the cleaned surface.

### 3.3. Roughness and Three-Dimensional Topography of the Surface of the Cleaned Material

In order to reuse the cleaned materials, the base metal should not be damaged, and the surface should meet some roughness requirements after laser cleaning. Therefore, the roughness and three-dimensional topography of the surface of the cleaned material were evaluated.

#### 3.3.1. The Effect of Laser X-Scanning Speed

**Figure 11:**
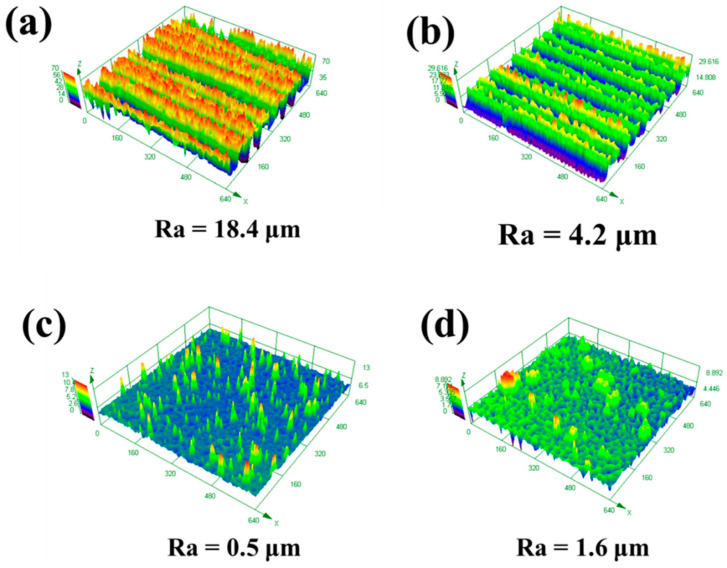
The effect of X-scanning speed on the three-dimensional morphology of the cleaned surface: (**a**) 500 mm/s (**b**) 1000 mm/s; (**c**) 1500 mm/s; (**d**) 2000 mm/s at a laser Y-moving speed of 7 mm/s.

Figure 11 shows the effect of the X-scanning speed on the three-dimensional morphology of the cleaned surface at a laser Y-moving speed of 7 mm/s. When the X-scanning speed was 500 mm/s, there were deep grooves on the surface of the sample, resulting in the surface roughness of 18.4 μm, as shown in Figure 11a. When the X-scanning speed increased to 1000 mm/s, the gully traces became shallower, causing a decrease in the roughness, as shown in Figure 11b. While, in case of X-scanning speed of 1500 mm/s, the traces of the ravines on the cleaned surface disappeared. In addition, a few pits and some small particles presented on the cleaned surface, the roughness of which was as low as 0.5 μm in Figure 11c. As can be seen in Figure 11d, at the X-scanning speed of 2000 mm/s, the size and number of the pits became larger and more, and the size of the convex particles changed to be larger, inducing the occurrence of the roughness of 1.6 μm.

As the X-scanning speed is low, the laser energy absorbed by the surface of the substrate is relatively larger. Once the paint layer is completely removed, the surface of the substrate will melt and ablate, and the traces will be deeper [16]. As the X-scanning speed increases, the spot overlap effect decreases. Energy absorbed by the material surface is lowered and the material surface is less damaged [26,27], leading to the small roughness of the surface. At the same time, most of the paint on the surface can be removed in this working condition. Meanwhile, the paint layer could not peel off the surface at a higher X-scanning speed.

#### 3.3.2. The Effect of Laser Y-Moving Speed

**Figure 12:**
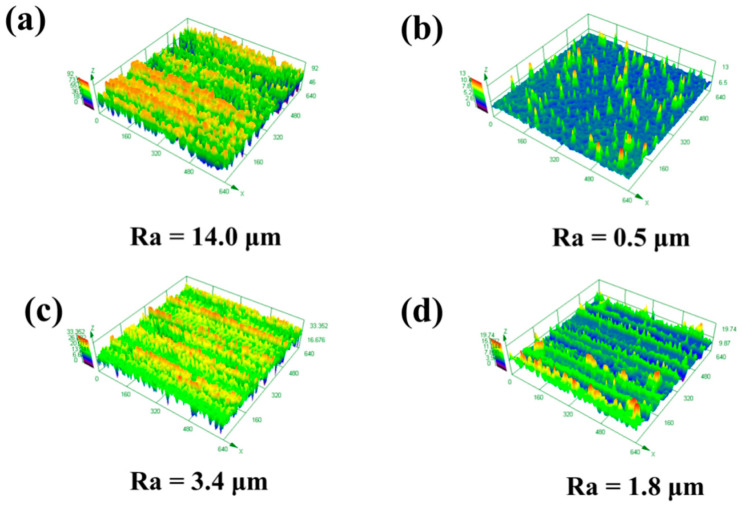
The effect of the Y-moving speed on the three-dimensional morphology of the cleaned surface: (**a**) 5 mm/s; (**b**) 7 mm/s; (**c**) 9 mm/s; (**d**) 11 mm/s at the laser X-scanning speed of 1500 mm/s.

**Figure 13:**
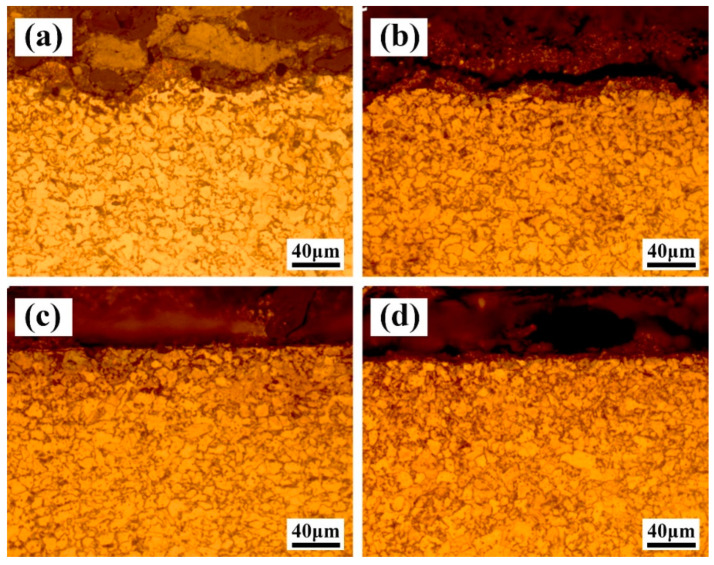
The effect of X-scanning speed on the metallographic structure of the cross-section of the Q345 steel (**a**) 500 mm/s; (**b**) 1000 mm/s; (**c**) 1500 mm/s; (**d**) 2000 mm/s at a Y-moving speed of 7 mm/s after laser cleaning.

Figure 12 shows the effect of the X-scanning speed on the three-dimensional morphology of the cleaned surface at a laser X-scanning speed of 1500 mm/s. When the Y-moving speed was 5 mm/s, deep grooves can be observed on the surface of the sample, resulting in the surface roughness of 14 μm, as shown in Figure 12a. As the Y-moving speed increased to 7 mm/s, deep grooves disappeared and a few pits presented on the cleaned surface, the roughness of which was as low as 0.5 μm in Figure 13b. While, in case of the Y-moving speeds of 9 mm/s and 11 mm/s, the traces of the ravines on the cleaned surface appeared again, and the roughness increased to 3.4 μm and 1.8 μm, respectively, as shown in Figure 12c,d.

A two-dimensional laser system was employed to clean the paint layer on the surface of the material, and thus the X-scanning speed of X direction and the Y-moving speed of Y direction plays an important role in the cleaning effect. When the X-scanning speed was too low, the substrate could be damaged. On the contrary, a significant number of the paint could not be cleaned clearly and remained on the surface. In addition, a linear residue paint could appear on the edge of the scanning path at a relatively high Y-moving speed [27]. Therefore, the clean effect was better on the condition of the Y-moving speed of 5 mm/s and the X-scanning speed of 1500 mm/s.

### 3.4. Microstructure of the Cleaned Region

During laser cleaning, except for the removal of the paint layer, the substrate could also be affected by the radiation of the laser under a certain condition. Thus, the microstructure and hardness of the cleaned Q345 steel are investigated too after laser cleaning. Figure 13 shows the effect of X-scanning speed on the metallographic structure of the cross-section of the steel at a Y-moving speed of 7 mm/s after laser cleaning. When the laser X-scanning speed was of 500 mm/s and 1000 mm/s, a fine grain layer around the laser scanning path could been seen in Figure 13a,b. Indicating that the X-scanning speed was 500 mm/s the fine grain layer thickness was bigger. However, no obvious fine grain layer was found at the X-scanning speeds of 1500 mm/s and 2000 mm/s in Figure 13c,d.

**Figure 14:**
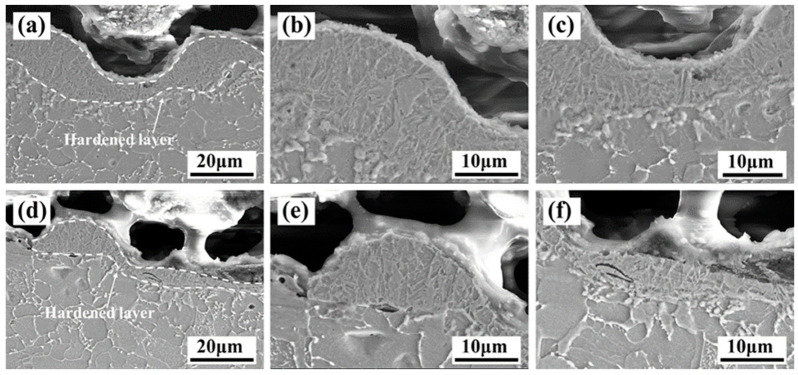
SEM image of the microstructure of the cross-section of the cleaned Q345 steel at a Y-moving speed of 7 mm/s and different X-scanning speeds after laser cleaning. (**a**) microstructure at 500 mm/s; (**b**) enlarged microstructure of the peak at 500 mm/s; (**c**) enlarged microstructure of the valley at 500 mm/s. (**d**) microstructure at 1000 mm/s; (**e**) enlarged microstructure of the peak at 1000 mm/s; (**f**) enlarged microstructure of the valley at 1000 mm/s.

Figure 14 reveals that the X-scanning speed had a greater influence on the formation of the microstructure. When the X-scanning speed was 500 mm/s, the fine grain layer was thicker at peak than valley, as shown in Figure 14a–c. The peak and valley of fine grain layer in case of the X-scanning speed of 1000 mm/s was given in Figure 14d–f, the fine grain layer became thinner by the comparison of those of 500 mm/s.

**Figure 15:**
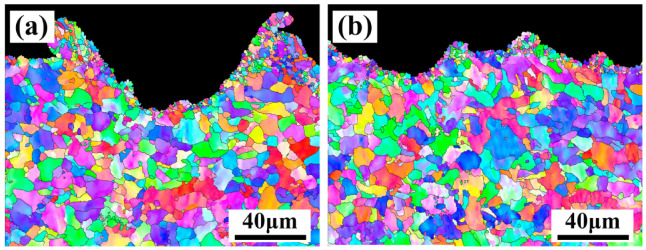
EBSD results at a Y-moving speed of 7 mm/s and different X-scanning speeds (**a**) 500 mm/s; (**b**) 1000 mm/s after laser cleaning.

The formation of the fine grain layer is mainly due to the combined effect of surface melting and shock waves. On the one hand, once the surface of the material absorbs laser energy, the temperature will rise rapidly to reach the melting point of the material, inducing to forming a molten state. It is known that the pulse laser has a shorter action time, the matrix will quickly cool and solidify to form a new structure [28,29,30]. On the other hand, the pulse laser will generate a high-pressure plasma shock wave after acting on the paint layer. When the shock wave pressure is greater than the elastic limit of the material, the material will undergo plastic deformation and the microstructure will change to form a fine grain layer [31,32]. In addition, the peak part belonged to the middle of two scanning path and accumulate more heat. Thus, the peak part absorbed more laser energy than the valley, resulting in the fine grain layer. In order to further confirm whether the grain size is changed, an EBSD test was performed. When the X-scanning speed of the material was 500 mm/s, more refined grains could be found in the fine grain layer compared with that of a X-scanning speed of 1000mm/s, as shown in Figure 15a,b.

**Figure 16:**
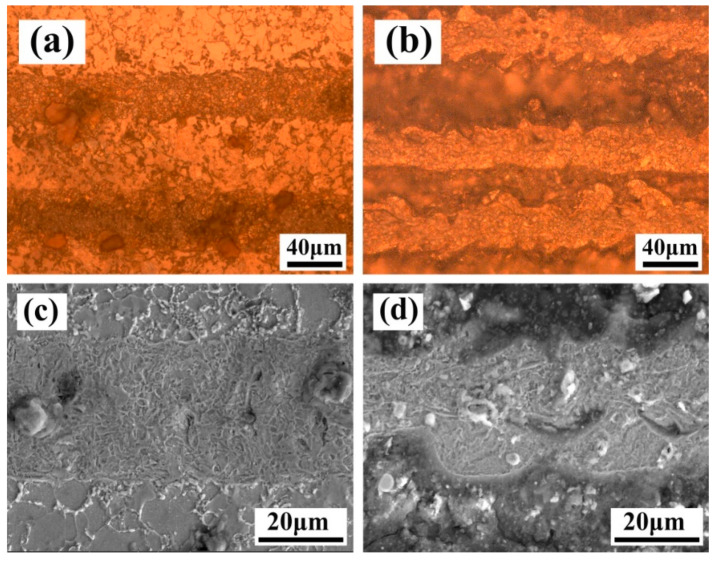
OM and SEM micrographs of the material surface after laser cleaning at different X-scanning speed. (**a**) 500 mm/s (OM); (**b**) 1000 mm/s (OM); (**c**) 500 mm/s (SEM); (**d**) 1000 mm/s (SEM).

The morphology of the material surface would also be investigated at the X-scanning speeds of 500 mm/s and 1000 mm/s. OM and SEM images of the morphologies are shown in Figure 16a–d, respectively. The microstructure along the scanning path of the light spot has been significantly refined. The width of the refined layer at a X-scanning speed of 500 mm/s was larger and the microstructure was finer than that at 1000 mm/s. At the low X-scanning speed, the radiation per unit area is greater on the surface of the material, leading to larger melting areas. In addition, the plasma shock wave formed after the evaporation of paint layer [26], the laser heat affected area is wider and the grains of which are also finer.

### 3.5. Hardness of the Fine Grain Layer after Cleaning

**Figure 17:**
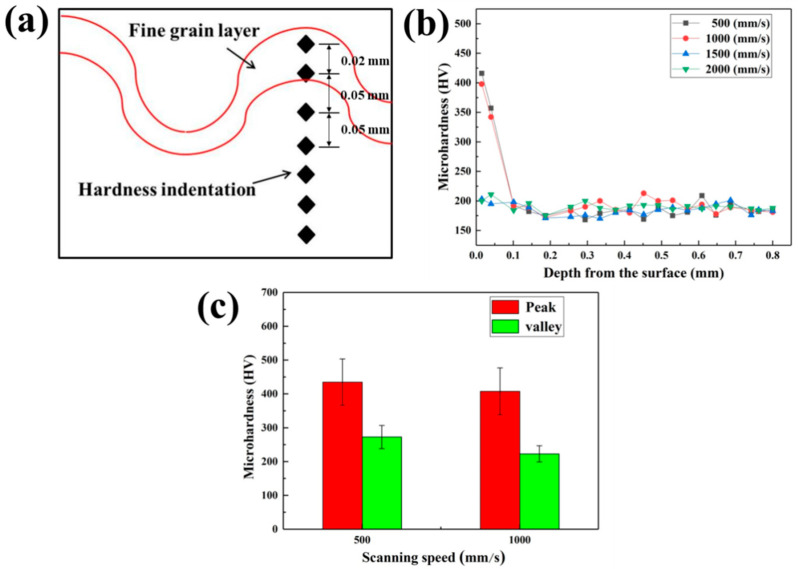
The result of the hardness of the material section. (**a**) The schematic diagram of the position of the hardness indentation; (**b**) The effect of X-scanning speed on the hardness of the material section; (**c**) The result of the hardness of the material section at different position.

A schematic diagram of the position of the hardness indentation is given in Figure 17a. From the surface, the first two hardness indentation was in the refined layer and others were in the base metal. In Figure 17b, when the X-scanning speeds were 500 mm/s and 1000 mm/s, the hardness of the first point from the surface is the highest, more than 400 HV. Then, the hardness of the second point decreased to about 350 HV. Meanwhile, in the case of higher X-scanning speeds, the hardness of the first two points from the surface was same as that of base metal, about 200 HV. Thus, it can be confirmed that the surface of the material produces a hardened layer at the low X-scanning speed after laser cleaning. The grain refinement makes the dislocation movement more difficult. Grain deformation and slippage becomes difficult, which can resist larger external pressure and lead to the increase of the surface micro-hardness [32,33].

In addition, the hardness of the first point of the peak and valley was compared at the X-scanning speeds of 500 mm/s and 1000 mm/s, as shown in Figure 17c. With the near-surface hardness of the material at a X-scanning speed of 500 mm/s, the hardness at the peak and valley was slightly higher than that at 1000 mm/s. Regardless which X-scanning speed, the hardness at the peak was higher than those at the valley. At the low X-scanning speed, the radiation per unit area is more on the surface of the material, leading to a rise in the temperature and the plasma shock wave. The formed crystal grains were smaller, and the movement of dislocations was blocked. Greater pressure was required to overcome it, resulting in higher hardness. When the laser spot acts on the surface of the material, the peak part was in the middle of the scanning path of the light spot, resulting in more heat accumulation effect. Therefore, the hardened layer produced was thicker, and the indentation can completely fall into the hardened layer, leading to greater hardness.

## 4. Conclusions

Laser technology was employed to study the effect of laser X-scanning speed and Y-moving speed on the cleaning effect of Q345 surface paint. The morphology, chemical composition, roughness, microstructure, and hardness of the cleaned material were studied. The conclusions are as follows.

(1) When the Y-moving speed was of 7 mm/s, the cleaned surface was damaged due to laser radiation of the relatively high energy at the X-scanning speeds of 500 mm/s and 1000 mm/s. Then, the content of Fe element on the cleaned surface increased to 89% and then decreased to about 80%. With increasing X-scanning speed from 500 mm/s up to 2000 mm/s, the roughness declined up to 0.5 μm and then had a little promotion.
(2) When the X-scanning speed was equal to 1500 mm/s, the amount of the residual paint on the cleaned surface tended to increase with increasing Y-moving speed from 5 mm/s to 11 mm/s. This is because the action time of the laser radiation on the surface became short. Meanwhile, the content of Fe element on the cleaned surface had a tendency of firstly decreasing and then remaining constant. Moreover, the roughness reached 0.5 μm at a Y-moving speed of 7 mm/s.
(3) Comprehensively, the cleaning effect of the surface paint could be better on the condition of a X-scanning speed of 1500 mm/s and a Y-moving speed of 7 mm/s during laser cleaning, since there was no damage, negligible residual paint, and the minimum roughness on the cleaned surface.
(4) A fine grain layer appeared on the cleaned surface after laser cleaning at the X-scanning speeds of 500 mm/s and 1000 mm/s, and a Y-moving speed of 7 mm/s. The maximum hardness of the fine grain layer was two times higher than the base metal, more than 400 HV. Moreover, the hardness of the peak of the fine grain layer was larger than those at the valley.

## Figures and Tables

**Table 1:** Chemical composition of the Q345.

C	Si	Mn	P	S	Cr	Mo	Ni	Al	Fe
0.21	0.12	0.96	<0.0005	0.0026	0.03	0.01	0.02	0.04	Bal.

**Table 2:** Main parameters of laser cleaning process.

Laser Main Parameters/Unit	Value
X-scanning speed/(mm/s) Y-moving speed/(mm/s)	500, 1000, 1500, 2000 5, 7, 9, 11
Average rated power/W	100
Single pulse energy/(mJ)	2
Repetition rate/(kHz)	100
Pulse width/(ns)	400
Spot size/(mm)	0.6
